# Mutation and Interaction Analysis of the Glycoprotein D and L and Thymidine Kinase of Pseudorabies Virus

**DOI:** 10.3390/ijms231911597

**Published:** 2022-09-30

**Authors:** Xue Li, Si Chen, Liying Zhang, Jiawei Zheng, Guyu Niu, Lin Yang, Xinwei Zhang, Linzhu Ren

**Affiliations:** College of Animal Sciences, Key Lab for Zoonoses Research, Ministry of Education, Jilin University, 5333 Xi’an Road, Changchun 130062, China

**Keywords:** pseudorabies virus (PRV, or Suid herpesvirus 1), glycoprotein, thymidine kinase (TK), nectin, heparan sulfate (HS)

## Abstract

Pseudorabies (also called Aujeszky’s disease) is a highly infectious viral disease caused by the pseudorabies virus (PRV, or Suid herpesvirus 1). Although the disease has been controlled by immunization with the PRV-attenuated vaccine, the emerging PRV variants can escape the immune surveillance in the vaccinated pig, resulting in recent outbreaks. Furthermore, the virus has been detected in other animals and humans, indicating cross-transmission of PRV. However, the mechanism of PRV cross-species transmission needs further study. In this study, we compared the amino acid sequences of glycoproteins (gD), gL, and thymidine kinase (TK) of PRV strains, human PRV hSD-1 2019 strain, and the attenuated strain Bartha-K61, followed by predication of their spatial conformation. In addition, the interactions between the viral gD protein and host nectin-1, nectin-2, and HS were also evaluated via molecular docking. The results showed that the amino acid sequence homology of the gD, gL, and TK proteins of hSD-1 2019 and JL-CC was 97.5%, 94.4%, and 99.1%, respectively. Moreover, there were mutations in the amino acid sequences of gD, gL, and TK proteins of hSD-1 2019 and JL-CC compared with the corresponding reference sequences of the Bartha strain. The mutations of gD, gL, and TK might not affect the spatial conformation of the protein domain but may affect the recognition of antibodies and antigen epitopes. Moreover, the gD protein of JL-CC, isolated previously, can bind to human nectin-1, nectin-2, and HS, suggesting the virus may be highly infectious and pathogenic to human beings.

## 1. Introduction

Pseudorabies (also called Aujeszky’s disease) is a highly infectious viral disease caused by the pseudorabies virus (PRV, or Suid herpesvirus 1), which is widely distributed and prevalent in the world and has caused significant economic losses to the world’s pig industry [1,2,3]. Furthermore, PRV can establish a lifelong latent infection in infected pigs but can be re-activated from latency after stress stimulation [4,5,6]. Therefore, latently infected pigs may become the source of re-emerging disease, which is one of the difficulties in eradicating PRV [4,5].

PRV is an enveloped, linear double-stranded DNA virus belonging to the *Varicellovirus* genus in the *Alphaherpesvirinae* subfamily of the *Herpesiviridae* family [1,2,7]. The viral genome is about 145 kb, which encodes 70–100 proteins, mainly capsid proteins, envelope proteins, tegument proteins, and enzymes [7]. Among these proteins, PRV thymidine kinase (TK), encoded by the viral *UL23* gene, is the critical virulence factor of PRV, which is also participates in re-activating the virus from the latent period and the neuro-invasiveness of PRV [8,9,10]. Furthermore, eleven glycoproteins, including gB, gC, gD, gE, gG, gH, gI, gK, gL, gM, and gN, and four transmembrane proteins, UL20, UL43, US9, and UL24, are found on the virion envelope [3]. During the entry, the viral gC, gB, gD, gH, and gL participate in the attachment and the membrane fusion [3]. Therefore, glycoproteins, such as gC, gB, gD, gE, gH, gL, and gIII, are the dominant antigens stimulating host innate immune responses [11,12]. Moreover, the gD and gL proteins are essential for virus infection [3,13]. For example, the viral gD on the virion surface can recognize and attach to immunoglobulin (Ig)-like cell adhesion molecules such as nectin-1, nectin-2, and heparan sulfate (HS) [14,15,16,17,18]. Notably, PRV gD can interact with both human and porcine nectin-1 with similar affinity [16].

The PRV natural attenuated vaccine strain Bartha-K61 was identified after multiple passages of a virulent field isolate in chicken cells and embryos [3]. Several independent mutations contributing to its attenuate were confirmed, including point mutations within UL21, a signal sequence mutation in the UL44 (gC) gene, and a 3 kb deletion encompassing US8 (gE), US9, and a large portion of US7 (gI) and US2 [3,19,20]. Therefore, the Bartha strain has been widely used in domestic pig farms since the 1990s [3]. However, since 2011, PRV infection cases have gradually increased in pig farms immunized with the gE-negative vaccine Bartha-K61 in China, which were associated with infection of PRV variants generated by recombination between PRV field isolates or field strains and vaccine strains [2,21,22,23,24]. Furthermore, more than 20 cases of PRV infections in humans have been reported, which are characterized by fever, seizures, human encephalitis, endophthalmitis, severe central nervous system symptoms, etc. [25,26,27]. These results indicate that PRV and its re-emerging variants may pose a significant threat to the pig industry and human beings. Therefore, it is necessary to compare the genetic variation of PRV from porcine and PRV isolated from humans. 

In this study, we compared and analyzed the genetic relationship and variation of gD, gL, and TK proteins of different PRV strains, followed by predication of their spatial conformation. In addition, the interactions between the viral gD protein and host nectin-1, nectin-2s, and HS were also evaluated via molecular docking. These results may provide a theoretical basis for exploring the mutations of gD, gL, and TK proteins of PRV variants and preventing and controlling PRV variants from infecting humans, domestic pigs, and other permissive animals. 

## 2. Results

### 2.1. The Amino Acid Sequences of PRV Strains in China Are Different from That Reported Abroad

**Figure 1:**
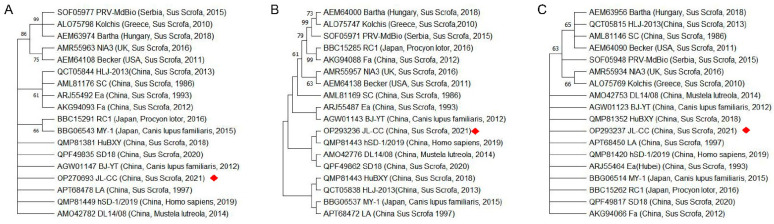
Phylogenetic tree based on the amino acid sequences of PRV. (**A**) gD, (**B**) gL, (**C**) TK. The red diamond indicates the PRV JL-CC strain, which was previously isolated from Jilin province, China [1]. The year, country, and host of isolates are indicated in the brackets.

Amino acid sequences of PRV gD, gL, and TK were analyzed, followed by the construction of phylogenetic trees. As shown in Figure 1, the amino acid sequences of gD, gL, and TK proteins of PRV isolates reported in China (JL-CC, LA, hSD-1 2019, DL14/08, BJ/YT, JL-CC, SD18, and BJ/YT strains) have low homology with those of isolates reported abroad, including the PRV strains Bartha, Kolchis, NIA3, Becker, RC1 and MdBio. Furthermore, the PRV isolates reported previously (Ea, Fa, SC) and PRV variants isolated in recent years (hSD-1 2019, DL14/08, BJ/YT, JL-CC, SD18, and BJ/YT) have relatively low homology of gD and gL proteins, but little differences between the TK proteins. These results suggest that the antigenic epitopes of PRV epidemic strains in China might be changed in recent years. In contrast, the virulence of PRV epidemic strains may not be weakened, which is one of the reasons for the decrease in the protection of the current PRV vaccine and the frequent outbreak of pseudorabies caused by the PRV variant.

### 2.2. Mutations in PRV gD and gL Proteins May Affect the Recognition of Antigenic Epitopes

As the PRV Bartha strain is a classic vaccine strain and PRV hSD-1 2019 is a human isolate [28], we selected the amino acid sequences of gD, gL, and TK proteins of PRV Bartha, hSD-1 2019, JL-CC for mutation analysis. The results showed that the amino acid sequence homology of gD, gL, and TK proteins of hSD-1 2019 and JL-CC were 97.5%, 94.4%, and 99.1%, respectively. Moreover, there were mutations in the amino acid sequences of the gD, gL, and TK proteins of hSD-1 2019 and JL-CC compared with the corresponding reference sequences of the Bartha strain (Table 1). In addition, the mutations of the gD (A69V, S82N), gL (T84N, I122V), and TK (T215V) proteins of hSD-1 2019 and JL-CC were identified in the interaction domains of the Herpesvirus glycoprotein D/GG/GX, Herpesvirus glycoprotein L family, and Thymidine kinase from herpesvirus, respectively (Table 1). 

Theoretically, these mutations could affect the function of the gD, gL, and TK proteins. Therefore, the T and B cell epitopes of gD, gL, and TK proteins of Bartha, PRV hSD-1 2019, and JL-CC were evaluated via the online software Pfam, ABCpred, and NetCTL. As expected, although these mutations do not affect T cell epitopes, the composition of B cell epitopes in PRV hSD-1 2019 and JL-CC was changed compared with that of the Bartha (Table 1). The B cell epitopes of the gD protein in PRV hSD-1 2019 and JL-CC were mutated from serine (S) to asparagine (N) at position 82 (S82N), respectively. Moreover, the mutations A45H and G62D were identified in the gL protein of PRV hSD-1 2019 and JL-CC, respectively. Therefore, the mutations of the gD (S82N) and gL proteins (A45H, G62D, T84N) in the PRV hSD-1 2019 and JL-CC strains may change the epitopes of gD and gL proteins, resulting in the shielding of neutralizing antibodies’ epitopes and the decrease in vaccine protection. However, these results still need to be verified by in vivo experiments.

### 2.3. The Mutations of gD, gL, and TK Proteins Did Not Affect Their Spatial Conformation but May Affect Their Function

**Figure 2:**
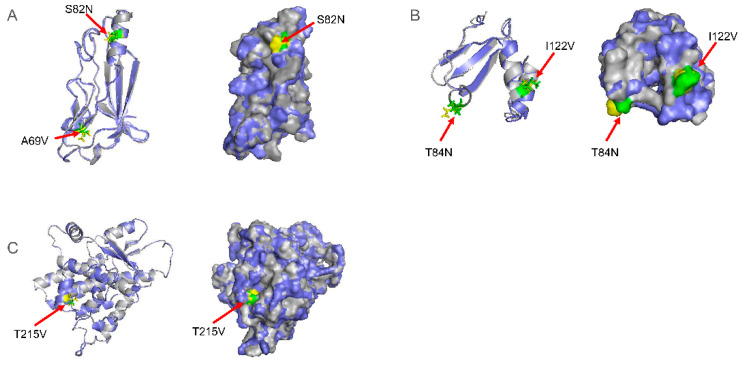
Predicated 3D structures of gD (**A**), gL (**B**), and TK (**C**) proteins. Bartha is marked as gray, and JL-CC is labeled by slate. Different amino acids of Bartha and JL-CC strains are highlighted in green and yellow, respectively.

To analyze whether gD, gL, and TK proteins’ mutations affect these proteins’ structure and function, we modeled the domains of gD, gL, and TK protein of Bartha, hSD-1 2019, and JL-CC strain. Since the protein sequences of hSD-1 2019 and JL-CC were completely identical, we only modeled the protein domains of Bartha and JL-CC to predict the effect of mutation on protein structure (Figure 2). For the gD protein, the RMSD value of Bartha and JL-CC/hSD-1 2019 was 0.225 Å. The RMSD value of the gL protein of Bartha and JL-CC/hSD-1 2019 was 0.696 Å. Meanwhile, the RMSD value of the TK protein of Bartha and JL-CC/hSD-1 2019 was 0.129 Å. The results indicated that the mutations of gD, gL, and TK proteins might not affect the spatial conformation of the protein domain.

Moreover, the mutations of these amino acids were further analyzed by Missence3D (Table 2). The results showed that the substitution did not alter the secondary structure of conformations of the ‘E’ (extended strand in parallel and/or anti-parallel β-sheet conformation), ‘T’ (hydrogen bonded turn), ‘G’ (3-turn helix), or ‘H’ (4-turn helix). Furthermore, the T84N mutation of the gL protein also showed only minor changes in crash, hydrophilicity, charge, and change exposure. The I122V mutation in the gL protein had little effect on these aspects. However, the A59V mutation in the gD protein altered the clash, hydrophilicity, charge, and cavity. The S82N mutation in gD protein mainly affected the hydrophilicity, charge, H-bond, and reduced exposure. For the TK protein, the T215V mutation altered the hydrophilicity, charge, and increased exposure. The amino acid mutations in gD, gL, and TK proteins did not destroy the structure and conformation of these proteins, which indicated that the protein structures of different PRV isolates are stable. However, the mutation changed the properties of amino acids, which may affect the functions of gD, gL, and TK proteins. At the same time, gD protein S82, and gL proteins A45, G62, and T84 were located in the linear epitopes of these proteins, which further indicates that the amino acid mutations at these sites may affect the recognition of antibodies and antigen epitopes. 

### 2.4. PRV JL-CC gD Protein Can Bind to Human Nectin-1, Nectin-2, and HS

**Figure 3:**
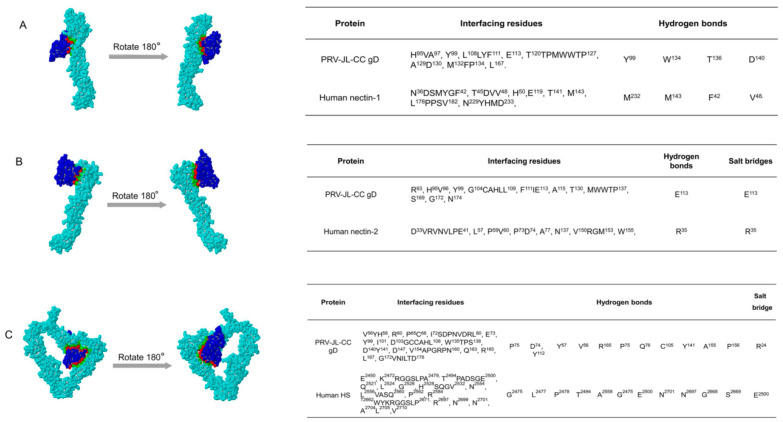
The structure and interface of PRV JL-CC gD with human nectin-1 (**A**), human nectin-2 (**B**) and human HS (**C**). The tables listed the interfacing residues and type of interaction. The gD protein of PRV JL-CC is marked in blue, and its binding site with nectin-1, nectin-2 and HS is marked with red; human nectin-1, nectin-2 and HS are marked with cyan, and its binding site with gD protein is labeled with green.

Nectin-1 and nectin-2, as gD receptors, are widely used for the cell entry of multiple alphaherpesviruses [14,16]. Since several groups reported cases of human infections with PRV, we further evaluated the binding affinity between the gD protein of PRV JL-CC strain with human nectin-1 and human nectin-2. The molecular docking results showed that the gD protein could interact with human nectin-1, nectin-2, and HS (Figure 3). Furthermore, multiple sites (residues) were identified in the gD protein, mediating the interaction between the gD protein and receptors. Most of these residues can form hydrogen bonds or salt bridges with the receptors to maintain the interaction. The residues Y99, W134, T136, and D140 of viral gD protein interact with the residues M232, M143, F42, and V48 of human nectin-1 via hydrogen bonds, respectively. The residue E113 of viral gD formed both hydrogen bond and salt bridges with the R35 of nectin-2. In the docking model between viral gD and HS, the residue R24 of viral gD and E2500 of HS forms five salt bridges, and 12 hydrogen bonds are predicated between gD and HS. These results suggest that the interactions between these sites play essential roles in the stability of the gD/HS complex.

## 3. Discussion

PRV is one of the most prevalent porcine viruses in the world. In addition, the virus can cross-transmit to other species, which has been reported in many animals, such as cats, dogs, cattle and wolves, and humans [10,25,26,27,29,30,31]. For example, neurological symptoms were observed in rhesus monkeys infected with PRV via intracerebral or intramuscular injection [32,33]. On the other hand, rhesus monkeys immunized with the herpes B virus vaccine can avoid the fatal consequences caused by PRV infection [32,33]. To date, although there is no direct evidence that PRV is infectious and pathogenic to humans, more than 20 cases of human infections have been identified as being related to PRV infection, characterized by fever, seizures, human encephalitis, endophthalmitis, severe central nervous system symptoms, etc. [25,26,27]. These results indicate that PRV could spill over to human beings. In this study, we compared the amino acid sequences of gD, gL, and TK proteins of the PRV strain JL-CC isolated previously and PRV hSD-1 2019 isolated from humans [28]. As a result, the amino acid sequence of gD, gL, and TK proteins of the PRV JL-CC strain is identical to that of the PRV hSD-1 2019 strain (Table 1), suggesting that the PRV strains may have the same infection ability in human beings. Furthermore, the gD protein of PRV JL-CC could interact with human nectin-1, nectin-2, and HS (Figure 3), suggesting that PRV JL-CC may also be highly infectious and pathogenic to human beings. Therefore, the interaction mechanism of PRV glycoproteins, such as gD and gL, with different receptors in different species to clarify the mechanism of PRV recognition and entry into the host is in progress.

The glycoproteins of PRV are essential to the viral infection and play important roles in virus replication, such as entry, release, transmission, immunomodulation, etc. [3,7,13,15]. For example, during the entry process, the viral gC, gB, gD, gH, and gL participate in the entry and fusion processes [3]. In addition, the gD protein binds to a specific receptor to stabilize virion–host interactions, a process necessary for virus entry [3,8,9]. After that, PRV gB, gH, and gL proteins mediate the fusion of the viral envelope and cytoplasmic membrane, allowing the virus to enter the cytoplasm [15]. Meanwhile, the gD protein is also a key target of host humoral and cellular immune responses, and most monoclonal antibodies to PRV gD exhibit high viral neutralization against PRV infection [34]. In this study, we found mutations in the amino acid sequences of gD, gL, and TK proteins of hSD-1 2019 and JL-CC compared with the reference sequences of the Bartha strain (Table 1). Furthermore, the amino acid mutations may affect the recognition of antibodies and antigen epitopes (Figure 2 and Table 2), which may lead to a decrease in the protection of the vaccine against PRV. These results suggest that the vaccines based on the Bartha strain might be less effective in preventing PRV JL-CC and other variants. Therefore, we must re-investigate the current PRV strains in China and then develop vaccines against the local prevalent strains to control the disease. 

PRV TK is one of the essential virulence factors that is also involved in replication in non-mitotic tissues, such as neurons [8,9,10]. Currently, most PRV vaccines are live-attenuated, based on the viral TK- and other glycoprotein-deleted strains [35,36,37,38]. 

For example, a gE/gI/TK-deleted live PRV vaccine can effectively cross-protect pigs against classical and variant PRV challenges [37]. In addition, recombinant feline herpesvirus type 1 (FHV-1) with TK-deleted was attenuated in cats [38]. However, another group found that TK deletion did not ultimately decrease the pathogenicity of PRV in rats and dogs [10,36]. These indicate that the virulence of PRV TK is different in different species, and the influence of TK deletion on the pathogenicity of PRV in other species still needs to be explored. Furthermore, compared with the Bartha strain, whether the mutation of the TK protein (such as T215N) in the PRV JL-CC strain affects its virulence needs further study.

## 4. Materials and Methods

### 4.1. Virus Strain Information and Multiple Alignments

The PRV strain JL-CC was previously isolated from Jilin province, China [1]. The GenBank ID of gD, gL, and TK proteins of the PRV JL-CC strain are OP270693, OP293236, and OP293237. In addition, information on other PRV strains isolated from different regions/countries was obtained from NCBI (Supplemental Table S1).

The amino acid sequences of gD, gL, and TK proteins of 17 PRV strains were collected from GenBank (Supplemental Table S1) to evaluate the genetic variation of PRV strains. The amino acid sequences of gD, gL, and TK proteins of PRV strain Bartha (GenBank No. MT468550) were used as the reference sequence, and multiple alignments were performed using ClustalW of Mega version 11.0 [39].

### 4.2. Phylogenetic Tree

The phylogenetic tree of gD, gL, and TK protein sequences was constructed to elucidate the relationship between PRV strains. Phylogenetic analysis was performed using Maximum Likelihood (ML) in Mega version 11.0 with a bootstrap of 1000 replicates [39].

### 4.3. Functional Domain and Epitope Analysis of gD, gL, and TK Proteins 

The B cell epitopes of the gD and gL proteins of the Bartha strain were predicted by the ABCpred online server (https://webs.iiitd.edu.in/raghava/abcpred/ABC_submission.html, accessed on 30 August 2022) [40]. In addition, the NetCTL-1.2 online server (https://services.healthtech.dtu.dk/service.php?NetCTL-1.2, accessed on 30 August 2022) was used to predict the T cell epitopes of the gD and gL proteins of Bartha strains. The domains of gD, gL, and TK proteins of Bartha strains were predicted by Pfam (http://pfam.xfam.org/, accessed on 30 August 2022). The T and B cell epitopes of gD, gL, and TK proteins of PRV hSD-1 2019 and JL-CC were compared with that of the Bartha.

### 4.4. Three-Dimensional (3D) Structures Modeling

Three-dimensional (3D) structures of the domains of gD, gL, and TK protein were predicted using the online software I-TASSER (https://seq2fun.dcmb.med.umich.edu/I-TASSER/, accessed on 30 August 2022) [41,42].

The 3D structure of nectin-1 (NP_002846.3) was constructed based on the 3D structure of Poliovirus receptor-related protein 1 (PDB ID: 4fmf.2.B). The 3D model of nectin-2 (NP_002847.1) was created based on the 3D form of Poliovirus receptor-related protein 1 (PDB ID: 3j8f.1.E) using the online program Swiss-Model (https://swissmodel.expasy.org/, accessed on 30 August 2022) according to the protocol described in the program [43]. 

The 3D structural alignment was performed by the online program Pymol 2.0. In addition, RMSD (Schrödinger, NY, USA) analysis for the structural alignment of viral proteins was conducted according to the protocol described by Souza et al. [40].

### 4.5. The Mutation Analysis of the gD, gL, and TK Proteins

The mutation analysis of amino acids of gD, gL, and TK was performed on the predicted structures using Missence3D (http://missense3d.bc.ic.ac.uk/~missense3d/, accessed on 30 August 2022) [44].

### 4.6. Molecular Docking

Molecular docking was conducted to analyze the interaction between PRV gD and human nectin-1, nectin-2, and heparan sulfate (HS) using the online ZDock server (https://zdock.umassmed.edu/, accessed on 30 August 2022). In addition, the interaction was analyzed via online PDBePISA (https://www.ebi.ac.uk/msd-srv/prot_int/cgi-bin/piserver, accessed on 30 August 2022). 

## 5. Conclusions

In conclusion, our study shows the differences in the epitopes of gD and gL proteins between the PRV JL-CC strain and the Bartha strain, which may significantly decrease the immunity of current vaccines against novel strains. However, these mutations did not disrupt the structure of proteins and may not significantly impact their function. On the other hand, mutations in the TK protein may alter the virulence of the strain, making infected people or animals more severely ill. Moreover, the PRV gD protein may bind to human nectin-1, nectin-2, and HS, reminding us to be vigilant about the transboundary transmission of PRV to humans, resulting in severe disease.

## Figures and Tables

**Table 1:** Mutations in gD, gL, and TK proteins of PRV hSD-1 2019 and JL-CC strains compared with that of the Bartha strain.

Protein	Domain/Family			T Cell Epitopes			B Cell Epitopes		
	Bartha	hSD-1/2019	JL-CC	Bartha	hSD-1/2019	JL-CC	Bartha	hSD-1/2019	JL-CC
gD	56–181	**A69V** **S82N**	**A69V** **S82N**	Y^35^TESWQLTL^43^, G^358^TAMGALLV^356^, H^230^REVVNYWY^238^			Q^76^VDRLLSEAV^86^, R^165^RLVSVDGVN^174^, I^122^FGRCRRRTT^131^, A^70^LISDPQVDR^79^, S^169^VDGVNILTD^178^	**S82N**	**S82N**
gL	70–138	**T84N** **I122V**	**T84N** **I122V**	V^86^PSVVVKPY^94^, M^83^TAVTSVVV^91^			H^58^PLLGLEPPV^67^ R^44^APRREELEW^53^	**A45H** **G62D**	**A45H** **G62D**
TK	10–278	**T215V**	**T215V**						

Note: The amino acid sequence of the Bartha strain is used as the reference sequence. The mutations of the corresponding sites in the hSD-1/2019 and JL-CC isolates are highlighted in bold. No mutation in the T cell epitope was detected.

**Table 2:** Mutation analysis of PRV gD, gL, and TK protein domains between Bartha and JL-CC strains.

	Clash	Buried Hydrophilic Introduced	Buried Charge Introduced and Switch	Secondary Structure Altered	Disallowed phi/psi	Buried Charge Replaced	Buried H-Bond Breakage	Cavity Altered	Buried/Exposed Switch
gD A59V	+++	+++	+++	NA	−−−	−−−	−−−	++−	+−−
gD S82N	+−−	++−	++−	NA	−−−	++−	++−	−−−	++−
gL T84N	+−−	+−−	+−−	NA	−−−	−−−	−−−	−−−	++−
gL I122V	+−−	−−−	−−−	NA	+−−	−−−	−−−	−−−	−−−
TK T215V	−−−	+++	++−	NA	−−−	++−	−−−	−−−	++−

Note: “+” means difference, and “−” means no difference. The number of “+” or “−” represents the quantity of different or similar, respectively.

## Data Availability

Not applicable.

## Supplementary Materials

The supporting information can be downloaded at: https://www.mdpi.com/article/10.3390/ijms231911597/s1.
