# Supplementary material for: Mutation and Interaction Analysis of the Glycoprotein D and L and Thymidine Kinase of Pseudorabies Virus
Source: Int J Mol Sci. 2022 Sep 30;23(19):11597. doi: 10.3390/ijms231911597 (PMC9570442; doi:10.3390/ijms231911597)
Supplement: Supplementary file 1 [file ijms-23-11597-s001.zip › ijms-1930566-supplementary.pdf]

Supplemental Table S1 Information of Pseudorabies virus isolates/strains obtained from NCBI.

Isolate/strain	Accession	Length	Country	Host	Collection Date	gD	gL	TK
Bartha	JF797217	137764	Hungary	Sus scrofa	2011-01	AEM63974	AEM64000	AEM63956
MY-1	AP018925	143277	Japan	Canis lupus familiaris	2015-03	BBG06543	BBG06537	BBG06514
BJ/YT	KC981239	120534	China	Canis lupus familiaris	2012-10	AGW01147	AGW01143	AGW01123
hSD-1/2019	MT468550	143905	China	Homo sapiens	2019	QMP81449	QMP81443	QMP81420
DL14/08	KU360259	143254	China	Mustela lutreola	2014-08	AMO42782	AMO42776	AMO42753
RC1	LC342744	141757	Japan	Procyon lotor	2016-02-10	BBC15291	BBC15285	BBC15262
SD18	MT949536	143418	China	Sus scrofa	2020-08	QPF49835	QPF49862	QPF49817
HuBXY/2018	MT468549	144795	China	Sus scrofa	2018	QMP81381	QMP81375	QMP81352
HLJ-2013	MK080279	142560	China	Sus scrofa	2013-08	QCT05844	QCT05838	QCT05815
Ea (Hubei)	KX423960	141818	China	Sus scrofa	1993	ARJ55492	ARJ5547	ARJ55464
LA	KU552118	141428	China	Sus scrofa	1997-03-01	APT68478	APT68472	APT68450
Fa	KM189913	141930	China	Sus scrofa	2012-08-06	AKG94093	AKG94088	AKG94066
NIA3	KU900059	142228	United Kingdom	Sus scrofa		AMR55963	AMR55957	AMR55934
SC	KT809429	142825	China	Sus scrofa	1986	AML81176	AML81169	AML81146
Becker	JF797219	141113	USA	Sus scrofa		AEM64108	AEM64138	AEM64090
PRV-MdBio	LT934125	142922	Serbia	Sus scrofa domesticus	2015-09	SOF05977	SOF05971	SOF05948
Kolchis	KT983811	141542	Greece	Sus scrofa domesticus	2010	ALO75798	ALO75773	ALO75769
